# Generational changes in self-reported infertility among migrants in Australia

**DOI:** 10.1016/j.jmh.2025.100385

**Published:** 2025-12-11

**Authors:** Jasmin Passet-Wittig, Ester Lazzari, Nadja Milewski

**Affiliations:** a Federal Institute for Population Research, Friedrich-Ebert-Allee 4, 65180 Wiesbaden, Germany; b University of Vienna (Wittgenstein Centre for Demography and Global Human Capital (IIASA, OeAW, University of Vienna)), 1010 Vienna, Austria

**Keywords:** Infertility, Reproductive health, Healthy migrant effect, Social inequalities, Australia

## Abstract

Knowledge about the perinatal and reproductive health of international migrants remains limited due to their marginalization in health and social-epidemiological research. We address this gap by analysing the prevalence of self-reported infertility among three migrant generations, migrants from different regions of origin, and comparing it to the majority of third-generation plus in Australia. We examine whether infertility risk differs across these groups and to what extent socio-demographic characteristics account for observed differences. The analysis draws on pooled data from the 2011, 2015, and 2019 waves of the Household, Income, and Labour Dynamics in Australia (HILDA) survey, the waves in which self-reported infertility was assessed. Average prevalence of current infertility is 11.8% among women and 7.5% among men. Multivariable findings from this study align with the healthy migrant hypothesis, which suggests that migrants tend to have a health advantage over the majority population at destination which is driven by selection into migration. Australia’s immigration policy contributes to such selection effects as it favours immigration of skilled and healthy individuals, who likely have a lower risk of infertility. The health advantage of the migrant generations 1 and 1.5 occurs for all origin groups except for migrants from English-speaking countries. This advantage diminishes for the second generation. In light of the high levels of self-reported infertility in this study, results suggest that Australia’s reproductive health policies need to address the specific needs of Australia’s increasingly diverse population.

## Highlights

• There is evidence for a healthy migrant effect for self-reported infertility in women and men.
• First- and 1.5-generation migrants report lower infertility than long-term residents.
• Migrants from English-speaking countries do not differ from long-term residents.
• The initial health advantage disappears in the second migrant generation.
• Australia’s selective, skill-based migration system is crucial for understanding this advantage.

## 1 Introduction

Women’s and men’s age at birth is increasing in most high-income countries and so is their risk of experiencing infertility. Thus, postponement of births contributes to the increasing relevance of infertility as a reproductive barrier. Current global prevalence of medical infertility is estimated at 12.6 % (Cox et al., 2022), indicating that infertility affects many individuals and couples at a given point in time. Medical infertility applies if a pregnancy is not achieved after at least one year of regular unprotected intercourse (Zegers-Hochschild et al., 2017). The experience of infertility can have important consequences for individuals or couples because an important life goal is at risk. Infertility has been identified as a key area of sexual and reproductive health and rights (SRHR) by the Guttmacher-Lancet Commission (Starrs et al., 2018). According to the commission, information and services for SRHR should be available to everyone in need irrespective of their ethnicity or socio-economic situation.

Many industrialised countries are characterized by their multi-ethnic population composition. International migrants and their descendants make up a significant share of the population. The number of migrants and their descendants is expected to increase in many of these countries in the future resulting from ongoing immigration and differences in demographic behaviour between migrant and majority groups. These trends increase population heterogeneity further, also in the so-called classic immigration countries of Northern America and Oceania. In this paper, we argue that it is important to consider infertility as a factor of reproductive health in migrant populations, particularly given the limited knowledge about the reproductive health needs of these groups, including the need for infertility services (Passet-Wittig and Greil, 2021), making it difficult to evaluate whether they receive the care that they need and how their access and use of infertility services can be improved (Atkin, 2009).

Migration and marginalization processes are selective. Thus, certain migrant groups may be disadvantaged while others are not or are even better off. As a consequence, it is not sufficient to summarizes all migrants in one category, because within-group variation may be disguised (e.g. (Köppen et al., 2021)). Recent studies on infertility and on other perinatal and reproductive health outcomes highlight the need to take a closer look at migrant generation and region of origin (Milewski et al., 2025; Väisänen et al., 2022; Milewski and Peters, 2014). Studies from the US and Europe show that migrant and ethnic minority groups often have worse outcomes in reproductive health and access to reproductive health care (Milewski et al., 2025; Jackson-Bey et al., 2021; Merkison et al., 2023; Brautsch et al., 2023). Overall, the evidence regarding the direction of the association is mixed, with variations depending on factors such as the specific outcome and ethnic minority group. For example, Latino women in the US have similar rates of low birth weight as white women, despite being, on average, more socio-economically disadvantaged—a factor that is typically a strong risk for low birth weight (Fuentes-Afflick et al., 1999). Furthermore, it is difficult to compare findings across countries as country context matters a lot. The composition of migrant populations differs between countries and so does regulation of access to perinatal and reproductive health care.

The focus of the current study is on Australia which has a long history of immigration and is still a major immigration country. As of 2021, 29 % of the population were born overseas (first generation migrants) and an additional 22 % were children of recent migrants (second generation migrants) (Australian Bureau of Statistics 2022). Of the rest, the majority are third-generation plus migrants, with the exception of the indigenous populations. Australia’s immigration policy is highly managed. It has a strong focus on skilled migration using a point-based system. Additional requirements also apply, including health and English proficiency, which are essential for visa eligibility.

Many studies find a health advantage of migrants in Australia (Kennedy et al., 2015; Renzaho, 2016; Huang et al., 2023), providing evidence for the existence of a healthy migrant effect (HME). Such studies focus on global health indicators such as life expectancy or dimensions of general health including chronic diseases, while perinatal and reproductive health conditions are seldom the focus of such studies. Health screening and selection via skills and education are important processes contributing to the HME. The first is important because general health and reproductive health are interrelated. Moreover, skilled migrants typically have higher education and higher social status in general, which is again linked to health because of healthier lifestyle, better access and navigation of the health system, and potentially more financial resources. Most epidemiological infertility research from Australia has not included migrants as distinct groups in their studies (Herbert et al., 2009a; Herbert et al., 2009b; Marino et al., 2011). A study on Australian couples’ infertility and contraceptive use which did not particularly focus on migrants shows that couples where both partners were born overseas have a lower likelihood of identifying as infertile (Lazzari et al., 2022). We extend on this study by taking a more nuanced view at migrants.

This study aims to answer two related research questions: 1) Do migrant groups differ from non-migrant Australians in their prevalence of self-reported infertility? 2) If so, to what extent do health and socio-demographic characteristics account for these differences? This is to our knowledge the first study to account for variation in the migrant group in a study of self-reported infertility by comparing 1st -, 1.5- and 2nd -generation migrants and across regions of origin with third-generation plus Australians. The latter term is used to refer to the majority group of those living in Australia with no recent own or parent’s immigration experience. In this study of migrant infertility, we chose to not include indigenous Australians, a distinct population with unique cultural, health, and socio-economic characteristics. Addressing the differences of this group would have exceeded the scope of this paper.

## 2 Materials and methods

### 2.1 Data

For the analysis this study uses data from the Household, Income and Labour Dynamics in Australia (HILDA) Survey (release 20.0) (Summerfield et al., 2022). The HILDA Survey is a longitudinal household survey of a national probability sample of Australian private households with yearly interviews. The HILDA Survey started in 2001 with 13,969 respondent interviews from 7682 households aged 15 and older. In 2011, in order to replenish the sample another 2153 households with 4009 respondents were added. Interviews are performed face-to-face. Special topics such as infertility and contraception are included in HILDA only in specific waves. The three most recent waves in which the question on self- reported infertility was asked are included in the analysis: waves 11, 15 and 19. We pool data from three waves to increase sample size for the analysis of migrant group differences in infertility, a rather infrequent event. Thus, one respondent can contribute up to three observations. Household response rates for 2011 were 71.6 % for households in the main sample and 69 % in the refreshment sample; in 2015 and in 2019 the overall response rate was 70.2 % and 66.4 % respectively (Summerfield et al., 2022).

The pooled data set consists of 52,679 observations (23,154 persons) from both sexes. We excluded Indigenous Australians from the sample, reducing the sample to 51,057 observations (22,333 persons). Furthermore, the sample was restricted to women and men in romantic relationships at time of interview (35,770 observations from 16,653 persons) because the dependent variable captures infertility at the couple-level. In a next step the age range was restricted for the analysis of infertility. Only women aged 18–44 and men aged 18–77 are included in the analysis, reducing sample size to 19,908 observations (10,946 persons). Respondents below 18 years are not regularly asked about infertility. Men 45+ years are included if their female partner is <45 years in wave 11 and <50 years in waves 15 and 19. Similarly, for female respondents in wave 11 the upper age limit of women receiving the infertility question was 44 years, while in later waves it was 49 years. In order to have an equal age-range across waves the lower age limit was applied. It is not possible to harmonize the upper age limit of female partners of male respondents in waves 15 and 19 to 44 years because their exact age is not available in the HILDA survey.

Next, 3755 observations were excluded because they were pregnant or sterilized at the time of the interview resulting in a reduced sample of 16,107 observations (9798 respondents). This was done because the infertility question was only asked to individuals who were neither pregnant nor sterilized. Another 2087 observations of the new refreshment sample in wave 11 were dropped because the infertility question was not asked to them during their first wave of participation. Importantly, respondents from the refreshment sample do contribute observations in later waves. Only for another 105 observations there is no information on the infertility question because they replied with don’t know or no answer. From the remaining 13,915 observations (8464 persons) another 271 (2.0 %) observations were deleted because they had missing values on independent variables. Each respondent contributes an average of 1.6 observations to this study. The final analytical sample consists of 7041 observations from 4223 men and 6603 observations from 4089 women.

### 2.2 Measures

The outcome in this analysis is a binary measure indicating the current experience of infertility in the couple. The HILDA self-reported infertility measure conditions on medical advice “Based on medical advice, do you know of any physical or health reason that would make it difficult for you (and/or your partner) to have children/more children?”. Answering options were “yes”, “no”, or “don’t know”. This measure is distinct from other self-reported infertility measures for which respondents are either queried directly about the experience of a twelve-month period of unprotected intercourse without becoming pregnant or they are asked on the subjective evaluation of their fertility. It is possible that some people might perceive infertility but have not (yet) received medical advice, which would imply that we are underestimating infertility. HILDA contains no objective information on medical infertility, that is whether respondents have unsuccessfully tried to get pregnant and we also do not have any information on whether they actually received a diagnosis. Thus, it is not possible to cross-validate the information. Other studies using HILDA data have used this measure (Lazzari et al., 2022), but we are not aware of other surveys using this question.

To construct migrant generation, information from various HILDA variables was combined: respondents’ and respondents’ parents’ country of birth, and the year respondents first came to Australia to live. Our variable consists of four categories. (1) Australian respondents who did not migrate themselves and whose parents did not migrate are categorized as “Third-generation plus Australians”. For international migrants we distinguish three groups based on their own and their parents’ migration history. Such a distinction is crucial to understand the extent to which their health is shaped by different socialization contexts, living conditions, and adaptation processes. (2) Adult migrants born outside of Australia who immigrated at age 18+ are categorized as “first-generation migrants”. (3) Migrants born abroad who moved to Australia as children before age 18 were categorized as “1.5-generation migrants”. (4) Those born in Australia to one or two immigrant parents were grouped as “second migrant generation”.

The same variables were used to identify the region of origin for first - and 1.5-generation migrants (combined due to sample size) and second-generation migrants. The following regions of origin are distinguished: English-speaking countries (ESC: USA, Canada, New Zealand, United Kingdom, Ireland, and Norfolk Island), Asia, and other origins. Regions of origin for the second migrant generation are assigned based on their parents’ country of birth. A region is assigned if both parents were born there or if one parent was born there and the other parent was Australian-born. Other is assigned for other regions of origin or if parents were born if different regions of origin.

Based on previous research, several variables were identified as moderators of self-reported infertility across minority groups, all of which may vary across the groups studied. This study considers the age composition of the groups. Education, defined by the highest educational level achieved, is used as an indicator of social status. A binary indicator of low fertility desire is included to account for the correlation of wanting to have a child and experiencing fertility problems (Passet-Wittig et al., 2020). This variable is based on a question on whether one would like to have more children in the future using a 11-point Linkert scale. Low desire is indicated by values 0 to 4 and medium/high desire by values 5 to 10. Differing patterns of family formation such as relationship status (married vs. cohabiting/dating) and age at first birth could also potentially contribute to understanding group differences. Further control variables are geographical area (major city vs. more remote areas) and survey year. Sample descriptions by migrant generation are available in Table 1 and by a combined indicator of migrant generation and region of origin in Table 2 (men) and Table 3 (women).

**Table 1:** Sample characteristics by migrant generation, men and women.

	Men				Women			
	3rd-gen. plus Australians	1st-gen. migrant	1.5-gen. migrant	2nd-gen. migrant	3rd-gen. plus Australians	1st-gen. migrant	1.5-gen. migrant	2nd-gen. migrant
N	3996 (56.8 %)	704 (10.0 %)	612 (8.7 %)	1729 (24.6 %)	3901 (59.1 %)	642 (9.7 %)	464 (7.0 %)	1596 (24.2 %)
Age group								
18–29	1669 (41.8 %)	77 (10.9 %)	184 (30.1 %)	650 (37.6 %)	2039 (52.3 %)	156 (24.3 %)	167 (36.0 %)	784 (49.1 %)
30–39	1297 (32.5 %)	265 (37.6 %)	238 (38.9 %)	601 (34.8 %)	1373 (35.2 %)	340 (53.0 %)	212 (45.7 %)	582 (36.5 %)
40–44	466 (11.7 %)	127 (18.0 %)	88 (14.4 %)	226 (13.1 %)	489 (12.5 %)	146 (22.7 %)	85 (18.3 %)	230 (14.4 %)
45+ (men only)	564 (14.1 %)	235 (33.4 %)	102 (16.7 %)	252 (14.6 %)				
Age at first birth								
Childless	1961 (49.1 %)	202 (28.7 %)	258 (42.2 %)	813 (47.0 %)	1952 (50.0 %)	223 (34.7 %)	199 (42.9 %)	813 (50.9 %)
Less than 25	474 (11.9 %)	55 (7.8 %)	78 (12.7 %)	181 (10.5 %)	719 (18.4 %)	107 (16.7 %)	74 (15.9 %)	236 (14.8 %)
25–34	1196 (29.9 %)	286 (40.6 %)	206 (33.7 %)	544 (31.5 %)	1088 (27.9 %)	264 (41.1 %)	168 (36.2 %)	473 (29.6 %)
35+	365 (9.1 %)	161 (22.9 %)	70 (11.4 %)	191 (11.0 %)	142 (3.6 %)	48 (7.5 %)	23 (5.0 %)	74 (4.6 %)
Fertility desire								
Medium or high	2543 (63.6 %)	359 (51.0 %)	396 (64.7 %)	1125 (65.1 %)	2706 (69.4 %)	347 (54.0 %)	289 (62.3 %)	1060 (66.4 %)
Low	1453 (36.4 %)	345 (49.0 %)	216 (35.3 %)	604 (34.9 %)	1195 (30.6 %)	295 (46.0 %)	175 (37.7 %)	536 (33.6 %)
Relationship status								
Married	1684 (42.1 %)	506 (71.9 %)	323 (52.8 %)	809 (46.8 %)	1608 (41.2 %)	459 (71.5 %)	237 (51.1 %)	687 (43.0 %)
Cohabiting or dating	2312 (57.9 %)	198 (28.1 %)	289 (47.2 %)	920 (53.2 %)	2293 (58.8 %)	183 (28.5 %)	227 (48.9 %)	909 (57.0 %)
Level of education								
Did not finish high-school	659 (16.5 %)	46 (6.5 %)	69 (11.3 %)	230 (13.3 %)	495 (12.7 %)	47 (7.3 %)	25 (5.4 %)	146 (9.1 %)
Diploma or certificate	2300 (57.6 %)	271 (38.5 %)	342 (55.9 %)	1016 (58.8 %)	2102 (53.9 %)	196 (30.5 %)	220 (47.4 %)	867 (54.3 %)
Bachelor degree	642 (16.1 %)	176 (25.0 %)	139 (22.7 %)	327 (18.9 %)	863 (22.1 %)	233 (36.3 %)	150 (32.3 %)	387 (24.2 %)
Grad. diploma, masters or doctorate	395 (9.9 %)	211 (30.0 %)	62 (10.1 %)	156 (9.0 %)	441 (11.3 %)	166 (25.9 %)	69 (14.9 %)	196 (12.3 %)
Labor force status								
Unemployed	401 (10.0 %)	58 (8.2 %)	54 (8.8 %)	161 (9.3 %)	857 (22.0 %)	196 (30.5 %)	115 (24.8 %)	365 (22.9 %)
Part-time	432 (10.8 %)	78 (11.1 %)	58 (9.5 %)	218 (12.6 %)	1340 (34.4 %)	175 (27.3 %)	166 (35.8 %)	542 (34.0 %)
Full-time, 35-<40 h	2515 (62.9 %)	425 (60.4 %)	392 (64.1 %)	1052 (60.8 %)	990 (25.4 %)	146 (22.7 %)	113 (24.4 %)	425 (26.6 %)
Full-time, ≥40 h	648 (16.2 %)	143 (20.3 %)	108 (17.6 %)	298 (17.2 %)	714 (18.3 %)	125 (19.5 %)	70 (15.1 %)	264 (16.5 %)
Region								
Major city	2376 (59.5 %)	599 (85.1 %)	483 (78.9 %)	1289 (74.6 %)	2297 (58.9 %)	543 (84.6 %)	396 (85.3 %)	1149 (72.0 %)
Regional and remote areas	1620 (40.5 %)	105 (14.9 %)	129 (21.1 %)	440 (25.4 %)	1604 (41.1 %)	99 (15.4 %)	68 (14.7 %)	447 (28.0 %)
Survey wave								
2011	1034 (25.9 %)	138 (19.6 %)	158 (25.8 %)	442 (25.6 %)	1088 (27.9 %)	149 (23.2 %)	146 (31.5 %)	450 (28.2 %)
2015	1485 (37.2 %)	300 (42.6 %)	221 (36.1 %)	642 (37.1 %)	1387 (35.6 %)	258 (40.2 %)	160 (34.5 %)	575 (36.0 %)
2019	1477 (37.0 %)	266 (37.8 %)	233 (38.1 %)	645 (37.3 %)	1426 (36.6 %)	235 (36.6 %)	158 (34.1 %)	571 (35.8 %)

Data: HILDA waves 11, 15 and 19. *N* = 7041 men; *N* = 6603 women.

**Table 2:** Sample characteristics by migrant generation & region of origin, men.

	3rd-gen. plus Australians	1st /1.5-gen. migrants, ESC	1st /1.5-gen. migrants, Asia	1st /1.5-gen. migrants, other	2nd-gen. migrants, ESC	2nd-gen. migrants, Asia	2nd-gen. migrants, other
N	3996 (56.8 %)	471 (6.7 %)	419 (6.0 %)	426 (6.1 %)	831 (11.8 %)	277 (3.9 %)	621 (8.8 %)
Age group							
18–29	1669 (41.8 %)	91 (19.3 %)	68 (16.2 %)	102 (23.9 %)	361 (43.4 %)	114 (41.2 %)	175 (28.2 %)
30–39	1297 (32.5 %)	156 (33.1 %)	184 (43.9 %)	163 (38.3 %)	279 (33.6 %)	104 (37.5 %)	218 (35.1 %)
40–44	466 (11.7 %)	80 (17.0 %)	76 (18.1 %)	59 (13.8 %)	84 (10.1 %)	29 (10.5 %)	113 (18.2 %)
45+	564 (14.1 %)	144 (30.6 %)	91 (21.7 %)	102 (23.9 %)	107 (12.9 %)	30 (10.8 %)	115 (18.5 %)
Age at first birth							
Childless	1961 (49.1 %)	171 (36.3 %)	133 (31.7 %)	156 (36.6 %)	408 (49.1 %)	156 (56.3 %)	249 (40.1 %)
Less than 25	474 (11.9 %)	61 (13.0 %)	19 (4.5 %)	53 (12.4 %)	109 (13.1 %)	30 (10.8 %)	42 (6.8 %)
25–34	1196 (29.9 %)	145 (30.8 %)	187 (44.6 %)	160 (37.6 %)	266 (32.0 %)	66 (23.8 %)	212 (34.1 %)
35+	365 (9.1 %)	94 (20.0 %)	80 (19.1 %)	57 (13.4 %)	48 (5.8 %)	25 (9.0 %)	118 (19.0 %)
Fertility desire							
Medium or high	2543 (63.6 %)	252 (53.5 %)	231 (55.1 %)	272 (63.8 %)	538 (64.7 %)	192 (69.3 %)	395 (63.6 %)
Low	1453 (36.4 %)	219 (46.5 %)	188 (44.9 %)	154 (36.2 %)	293 (35.3 %)	85 (30.7 %)	226 (36.4 %)
Relationship status							
Married	1684 (42.1 %)	228 (48.4 %)	334 (79.7 %)	267 (62.7 %)	338 (40.7 %)	121 (43.7 %)	350 (56.4 %)
Cohabiting or dating	2312 (57.9 %)	243 (51.6 %)	85 (20.3 %)	159 (37.3 %)	493 (59.3 %)	156 (56.3 %)	271 (43.6 %)
Level of education							
Did not finish high-school	659 (16.5 %)	66 (14.0 %)	18 (4.3 %)	31 (7.3 %)	129 (15.5 %)	26 (9.4 %)	75 (12.1 %)
Diploma or certificate	2300 (57.6 %)	261 (55.4 %)	119 (28.4 %)	233 (54.7 %)	483 (58.1 %)	149 (53.8 %)	384 (61.8 %)
Bachelor degree	642 (16.1 %)	81 (17.2 %)	143 (34.1 %)	91 (21.4 %)	142 (17.1 %)	76 (27.4 %)	109 (17.6 %)
Grad. diploma, masters or doctorate	395 (9.9 %)	63 (13.4 %)	139 (33.2 %)	71 (16.7 %)	77 (9.3 %)	26 (9.4 %)	53 (8.5 %)
Labor force status							
Unemployed	401 (10.0 %)	36 (7.6 %)	30 (7.2 %)	46 (10.8 %)	80 (9.6 %)	27 (9.7 %)	54 (8.7 %)
Part-time	432 (10.8 %)	42 (8.9 %)	44 (10.5 %)	50 (11.7 %)	117 (14.1 %)	37 (13.4 %)	64 (10.3 %)
Full-time, 35-<40 h	2515 (62.9 %)	302 (64.1 %)	251 (59.9 %)	264 (62.0 %)	490 (59.0 %)	165 (59.6 %)	397 (63.9 %)
Full-time, ≥40 h	648 (16.2 %)	91 (19.3 %)	94 (22.4 %)	66 (15.5 %)	144 (17.3 %)	48 (17.3 %)	106 (17.1 %)
Region							
Major city	2376 (59.5 %)	341 (72.4 %)	393 (93.8 %)	348 (81.7 %)	583 (70.2 %)	238 (85.9 %)	468 (75.4 %)
Regional and remote areas	1620 (40.5 %)	130 (27.6 %)	26 (6.2 %)	78 (18.3 %)	248 (29.8 %)	39 (14.1 %)	153 (24.6 %)
Survey wave							
2011	1034 (25.9 %)	120 (25.5 %)	73 (17.4 %)	103 (24.2 %)	205 (24.7 %)	61 (22.0 %)	176 (28.3 %)
2015	1485 (37.2 %)	183 (38.9 %)	178 (42.5 %)	160 (37.6 %)	312 (37.5 %)	99 (35.7 %)	231 (37.2 %)
2019	1477 (37.0 %)	168 (35.7 %)	168 (40.1 %)	163 (38.3 %)	314 (37.8 %)	117 (42.2 %)	214 (34.5 %)

Notes: ESC = English-speaking countries. Data: HILDA waves 11, 15 and 19. *N* = 7041 men.

**Table 3:** Sample characteristics by migrant generation & region of origin, women.

	3rd-gen. plus Australians	1st /1.5-gen. migrants, ESC	1st /1.5-gen. migrants, Asia	1st /1.5-gen. migrants, other	2nd-gen. migrants, ESC	2nd-gen. migrants, Asia	2nd-gen. migrants, other
N	3901 (59.1 %)	281 (4.3 %)	504 (7.6 %)	321 (4.9 %)	768 (11.6 %)	263 (4.0 %)	565 (8.6 %)
Age group							
18–29	2039 (52.3 %)	91 (32.4 %)	130 (25.8 %)	102 (31.8 %)	409 (53.3 %)	137 (52.1 %)	238 (42.1 %)
30–39	1373 (35.2 %)	129 (45.9 %)	271 (53.8 %)	152 (47.4 %)	260 (33.9 %)	98 (37.3 %)	224 (39.6 %)
40–44	489 (12.5 %)	61 (21.7 %)	103 (20.4 %)	67 (20.9 %)	99 (12.9 %)	28 (10.6 %)	103 (18.2 %)
Age at first birth							
Childless	1952 (50.0 %)	118 (42.0 %)	173 (34.3 %)	131 (40.8 %)	406 (52.9 %)	173 (65.8 %)	234 (41.4 %)
Less than 25	719 (18.4 %)	55 (19.6 %)	70 (13.9 %)	56 (17.4 %)	119 (15.5 %)	17 (6.5 %)	100 (17.7 %)
25–34	1088 (27.9 %)	82 (29.2 %)	234 (46.4 %)	116 (36.1 %)	216 (28.1 %)	62 (23.6 %)	195 (34.5 %)
35+	142 (3.6 %)	26 (9.3 %)	27 (5.4 %)	18 (5.6 %)	27 (3.5 %)	11 (4.2 %)	36 (6.4 %)
Fertility desire							
Medium or high	2706 (69.4 %)	162 (57.7 %)	281 (55.8 %)	193 (60.1 %)	530 (69.0 %)	194 (73.8 %)	336 (59.5 %)
Low	1195 (30.6 %)	119 (42.3 %)	223 (44.2 %)	128 (39.9 %)	238 (31.0 %)	69 (26.2 %)	229 (40.5 %)
Relationship status							
Married	1608 (41.2 %)	129 (45.9 %)	382 (75.8 %)	185 (57.6 %)	306 (39.8 %)	99 (37.6 %)	282 (49.9 %)
Cohabiting or dating	2293 (58.8 %)	152 (54.1 %)	122 (24.2 %)	136 (42.4 %)	462 (60.2 %)	164 (62.4 %)	283 (50.1 %)
Level of education							
Did not finish high-school	495 (12.7 %)	21 (7.5 %)	40 (7.9 %)	11 (3.4 %)	70 (9.1 %)	12 (4.6 %)	64 (11.3 %)
Diploma or certificate	2102 (53.9 %)	127 (45.2 %)	149 (29.6 %)	140 (43.6 %)	441 (57.4 %)	123 (46.8 %)	303 (53.6 %)
Bachelor degree	863 (22.1 %)	87 (31.0 %)	192 (38.1 %)	104 (32.4 %)	166 (21.6 %)	84 (31.9 %)	137 (24.2 %)
Grad. diploma, masters or doctorate	441 (11.3 %)	46 (16.4 %)	123 (24.4 %)	66 (20.6 %)	91 (11.8 %)	44 (16.7 %)	61 (10.8 %)
Labor force status							
Unemployed	857 (22.0 %)	62 (22.1 %)	157 (31.2 %)	92 (28.7 %)	168 (21.9 %)	41 (15.6 %)	156 (27.6 %)
Part-time	1340 (34.4 %)	102 (36.3 %)	134 (26.6 %)	105 (32.7 %)	262 (34.1 %)	82 (31.2 %)	198 (35.0 %)
Full-time, 35-<40 h	990 (25.4 %)	66 (23.5 %)	121 (24.0 %)	72 (22.4 %)	206 (26.8 %)	79 (30.0 %)	140 (24.8 %)
Full-time, ≥40 h	714 (18.3 %)	51 (18.1 %)	92 (18.3 %)	52 (16.2 %)	132 (17.2 %)	61 (23.2 %)	71 (12.6 %)
Region							
Major city	2297 (58.9 %)	217 (77.2 %)	449 (89.1 %)	273 (85.0 %)	500 (65.1 %)	215 (81.7 %)	434 (76.8 %)
Regional and remote areas	1604 (41.1 %)	64 (22.8 %)	55 (10.9 %)	48 (15.0 %)	268 (34.9 %)	48 (18.3 %)	131 (23.2 %)
Survey wave							
2011	1088 (27.9 %)	93 (33.1 %)	110 (21.8 %)	92 (28.7 %)	219 (28.5 %)	68 (25.9 %)	163 (28.8 %)
2015	1387 (35.6 %)	98 (34.9 %)	200 (39.7 %)	120 (37.4 %)	272 (35.4 %)	89 (33.8 %)	214 (37.9 %)
2019	1426 (36.6 %)	90 (32.0 %)	194 (38.5 %)	109 (34.0 %)	277 (36.1 %)	106 (40.3 %)	188 (33.3 %)

Notes: ESC = English-speaking countries. Data: HILDA waves 11, 15 and 19. *N* = 6603 women.

### 2.3 Statistical analyses

For descriptive purposes, we show weighted current prevalence rates of self-reported infertility with 95 % confidence intervals by migrant generation and a combined indicator of migrant generation and region of origin. Binary logistic regression models are used to study the occurrence of self-reported infertility among the three migrant generations compared to third-generation plus Australians. Results from logistic regression are presented using average marginal effects (AME) with 95 % confidence intervals. AME are defined as an average effect of an independent variable on the probability of people reporting infertility which is based on the observed values of each person on all other variables in the model. AME have the advantage of being easily comparable across models (which is not the case for logit coefficients or odds ratios) and can be interpreted as predicted probabilities (Mood, 2010). Separate analyses are performed for women and men. Following recommendations for the analysis of pooled panel data with multiple observations per respondent, we estimate robust standard errors to account for multiple observations per person. All analyses are conducted using Stata 18 SE.

## 3 Results

### 3.1 Analyses by migrant generation

**Fig. 1:**
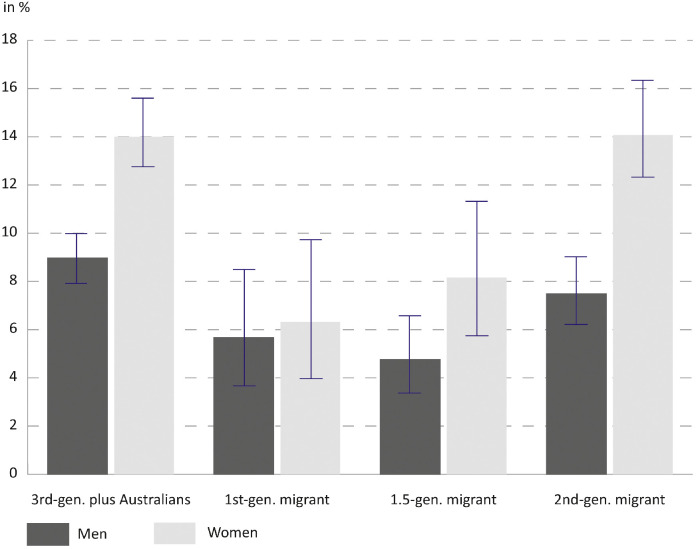
Prevalence of self- reported infertility by migrant generation (in %) with 95 % confidence intervals. Data: HILDA waves 11, 15 and 19. *N* = 7041 men; *N* = 6603 women; weighted.

Fig. 1 shows the weighted prevalence of self-reported infertility by migrant generation. The overall average prevalence for women is 11.8 %, which is 4.3 percentage points higher than the average for men. Among women, 1st and 1.5-generation migrants have lower rates of self- reported infertility than third-generation plus Australians. Prevalence increases from first-generation migrants to 1.5-generation migrants to second-generation migrants. Among men prevalence is highest for long-term residents with 9.0 %. For men, differences in prevalence rates between migrant generations are much less pronounced than for women.

In Table 4 results from logistic regression models are shown for men and women. In the first model we control only for age group because age is so central for infertility risk and also because first and 1.5 generation migrants are on average older than third-generation plus Australians (see Table 1). For first and 1.5 generation migrant men and women there is a statistically significant lower risk for self-reported infertility in comparison to the reference group. In adjusted models for women the risk of infertility is still decreased by 7.2 percentage points for first generation migrations and 5.5 percentage points for 1.5 generation migrants, which is almost twice the effect size of men.

**Table 4:** Average marginal effects (AME) with 95 % confidence intervals (CI95) for self-reported infertility according to migrant generation, men & women.

	Men		Women	
	Model 1	Model 2	Model 1	Model 2
	AME [CI95]	AME [CI95]	AME [CI95]	AME [CI95]
Migrant generation (ref. 3rd-gen. plus Australians)				
1st-gen. migrants	**−0.035**	**−0.035**	**−0.072**	**−0.072**
	[−0.057, −0.013]	[−0.059, −0.012]	[−0.097, −0.047]	[−0.098, −0.047]
1.5-gen. migrants	**−0.031**	**−0.031**	**−0.058**	**−0.055**
	[−0.056, −0.005]	[−0.057, −0.005]	[−0.089, −0.028]	[−0.087, −0.024]
2nd-gen. Migrants	−0.009	−0.010	0.006	0.008
	[−0.028, 0.009]	[−0.029, 0.009]	[−0.019, 0.030]	[−0.017, 0.032]
Age group (ref. 18–29)				
30–39	**0.035**	**0.036**	**0.047**	**0.062**
	[0.019, 0.050]	[0.018, 0.053]	[0.028, 0.065]	[0.040, 0.084]
40–44	**0.054**	**0.061**	**0.077**	**0.111**
	[0.031, 0.077]	[0.033, 0.090]	[0.049, 0.105]	[0.072, 0.149]
45+ (men only)	**0.074**	**0.072**		
	[0.049, 0.098]	[0.043, 0.101]		
Age at first birth (ref. childless)				
Less than 25		0.007		−0.006
		[−0.025, 0.038]		[−0.041, 0.028]
25–34		**−0.037**		**−0.054**
		[−0.058, −0.016]		[−0.082, −0.026]
35+		−0.009		−0.001
		[−0.038, 0.021]		[−0.049, 0.047]
Low fertility desire (ref. medium/high)		**−0.015**		**−0.034**
		[−0.031, 0.001]		[−0.055, −0.013]
Cohabiting or dating (ref. married)		**−0.047**		**−0.037**
		[−0.066, −0.027]		[−0.059, −0.015]
Level of education (ref. did not finish high-school)				
Diploma or certificate		−0.014		−0.021
		[−0.037, 0.010]		[−0.055, 0.012]
Bachelor degree		−0.025		**−0.043**
		[−0.054, 0.003]		[−0.080, −0.006]
Grad. diploma, masters or doctorate		−0.025		−0.034
		[−0.055, 0.006]		[−0.076, 0.007]
Labor force status (ref. unemployed)				
Part-time		**−0.035**		−0.019
		[−0.069, −0.002]		[−0.044, 0.005]
Full-time, 35-<40 h		**−0.033**		−0.024
		[−0.061, −0.005]		[−0.052, 0.003]
Full-time, ≥40 h		−0.029		**−0.031**
		[−0.060, 0.003]		[−0.060, −0.003]
Regional and remote areas (ref. major city)		0.009		0.003
		[−0.008, 0.026]		[−0.018, 0.023]
Survey wave (ref. 2011)				
2015		0.006		**0.019**
		[−0.009, 0.021]		[0.002, 0.036]
2019		**0.017**		**0.036**
		[0.000, 0.033]		[0.016, 0.055]
Number of observations	7041	7041	6603	6603

Notes: Statistically significant associations are highlighted in bold. Results from logistic regression of self-reported infertility.

Data: HILDA waves 11, 15 and 19.

Despite most of the moderating variables being significantly associated with infertility risk, the predicted probabilities for migrant generation are largely unaffected by their inclusion (Model 2 vs. Model 1). Associations broadly reflect what is known from the literature. The risk of infertility increases with age for men and women. Parents have a lower risk compared to childless women and men. Those with a medium or high fertility desire and those who are married are at a higher risk of reporting infertility. With increasing level of education, the infertility risk decreases, but only for women with a bachelor’s degree there is a significant negative association. In contrast, for labour force status we see that being employed is associated with a lower risk compared to being unemployed. Living in a regional or remote area is not associated with infertility risk.

### 3.2 Analyses by region of origin

**Fig. 2:**
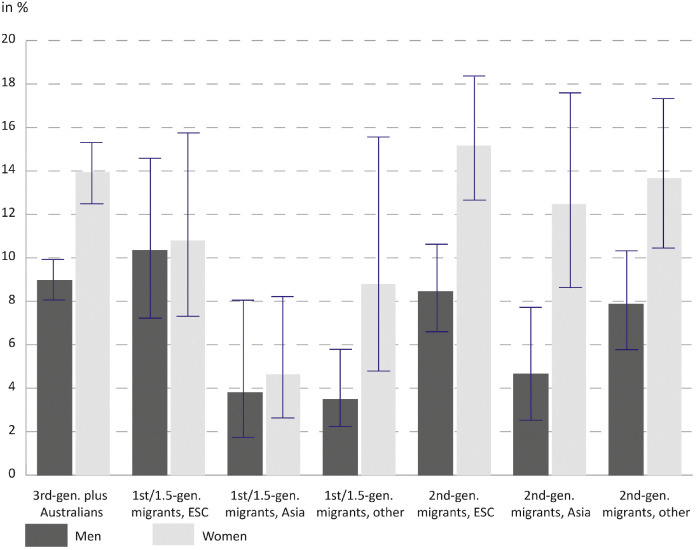
Prevalence of self- reported infertility for 1/1.5 generation migrants and second-generation migrants by region of origin (in %). Notes: ESC = English-speaking countries. Data: HILDA waves 11, 15 and 19. *N* = 7041 men; *N* = 6603 women; weighted.

Fig. 2 shows prevalence rates for migrants (generations 1 and 1.5 combined) and the second migrant generation by their region of origin. It shows that migrant women and men from Asia and second-generation women from Asia are by far the groups with the lowest rates of self-reported infertility. Among the second migrant generation sex differences are elevated with women indicating considerably higher prevalence rates than men.

Results in Table 5 suggest that the migrants from Asian countries and the heterogenous group of other origins are the driving forces behind the lower infertility risk of first and 1.5 generations. Migrants from English-speaking countries have similar (men) or higher (women) prevalence of self-reported infertility than third-generation plus Australians (see Fig. 2), but in the multivariable analyses this difference to non-migrant Australians is not statistically significant. Again, inclusion of potential moderators does not change any of the associations of the combined indicator of migrant generation and region of origin with infertility risk. Associations for control variables are very similar to those from the analysis of migrant generation.

**Table 5:** Average marginal effects (AME) with 95 % confidence intervals (CI95) for self-reported infertility according to migrant generation & region of origin, men & women.

	Men		Women	
	Model 1	Model 2	Model 1	Model 2
	AME [CI95]	AME [CI95]	AME [CI95]	AME [CI95]
Migrant generation & region of origin (ref. 3rd-gen. plus Australians)				
1st /1.5-gen. migrants, ESC	0.007	0.009	−0.02	−0.016
	[−0.026, 0.041]	[−0.025, 0.043]	[−0.063, 0.024]	[−0.060, 0.029]
1st /1.5-gen. migrants, Asia	**−0.057**	**−0.059**	**−0.088**	**−0.089**
	[−0.079, −0.036]	[−0.081, −0.037]	[−0.114, −0.062]	[−0.115, −0.063]
1st /1.5-gen. migrants, other	**−0.055**	**−0.057**	**−0.073**	**−0.073**
	[−0.078, −0.031]	[−0.081, −0.033]	[−0.105, −0.041]	[−0.105, −0.041]
2nd-gen. migrants, ESC	0.002	0.003	0.023	0.025
	[−0.024, 0.027]	[−0.023, 0.029]	[−0.011, 0.056]	[−0.009, 0.059]
2nd-gen. migrants, Asia	−0.031	−0.033	−0.006	−0.003
	[−0.070, 0.008]	[−0.072, 0.006]	[−0.056, 0.044]	[−0.054, 0.048]
2nd-gen. migrants, other	−0.015	−0.018	−0.012	−0.011
	[−0.040, 0.011]	[−0.043, 0.007]	[−0.047, 0.023]	[−0.046, 0.024]
Age group (ref. 18–29)				
30–39	**0.035**	**0.035**	**0.048**	**0.062**
	[0.020, 0.051]	[0.018, 0.053]	[0.029, 0.066]	[0.040, 0.084]
40–44	**0.054**	**0.060**	**0.077**	**0.111**
	[0.031, 077]	[0.032, 0.089]	[0.049, 0.105]	[0.072, 0.149]
45+ (men only)	**0.072**	**0.068**		
	[0.048, 0.096]	[0.040, 0.097]		
Age at first birth (ref. childless)				
Less than 25		0.007		−0.007
		[−0.024, 0.038]		[−0.041, 0.027]
25–34		**−0.036**		**−0.053**
		[−0.057, −0.016]		[−0.081, −0.025]
35+		−0.008		−0.003
		[−0.038, 0.021]		[−0.051, 0.044]
Low fertility desire (ref. medium/high)		−0.016		**−0.034**
		[−0.032, 0.000]		[−0.055, −0.013]
Cohabiting or dating (ref. married)		**−0.051**		**−0.039**
		[−0.070, −0.031]		[−0.061, −0.017]
Level of education (ref. did not finish high-school)				
Diploma or certificate		−0.012		−0.022
		[−0.036, 0.011]		[−0.056, 0.011]
Bachelor degree		−0.022		**−0.043**
		[−0.050, 0.006]		[−0.080, −0.006]
Grad. diploma, masters or doctorate		−0.020		−0.035
		[−0.050, 0.010]		[−0.077, 0.007]
Labor force status (ref. unemployed)				
Part-time		**−0.037**		−0.021
		[−0.071, −0.003]		[−0.046, 0.003]
Full-time, 35-<40 h		**−0.036**		−0.025
		[−0.064, −0.007]		[−0.053, 0.002]
Full-time, ≥40 h		−0.031		**−0.033**
		[−0.063, 0.001]		[−0.062, −0.004]
Regional and remote areas (ref. major city)		0.006		0.000
		[−0.011, 0.022]		[−0.020, 0.020]
Survey wave (ref. 2011)				
2015		0.006		**0.020**
		[−0.009, 0.020]		[0.003, 0.037]
2019		**0.017**		**0.037**
		[0.001, 0.033]		[0.017, 0.056]
Number of observations	7041	7041	6603	6603

Notes: ESC = English-speaking countries. Statistically significant associations are highlighted in bold. Results from logistic regression of self-reported infertility.

Data: HILDA waves 11, 15 and 19.

### 3.3 Data quality and robustness

The share of migrants in the HILDA data was compared with data from the population statistics for 2001, which was when the original sample for HILDA was drawn. The analytical sample (see Tables 2 and Tables 3, second row) reflects the composition of the Australian population of 2001 very well: migrant generations 1 and 1.5 combined (23 %), and individuals of the second migrant generation (25 %) (Australian Bureau of Statistics 2004).

The authors conducted several additional analyses as robustness checks. First, this paper focussed on women and men in a partnership because the outcome variable captures the experience of self-reported infertility in the couple. One concern among the authors was whether the results would hold in the full sample that included singles as well. Conclusions from the analysis of a sample which included not only partnered, but also single people are virtually the same. Second, using data only from waves 15 and 19 allows to study women between 18 and 49 years, rather than between 18 and 44 as in the analyses above. Results were again very similar with all substantial conclusions remaining valid. Thirdly, we also checked whether and how the definition of migrant generation would affect the infertility risk. This concerned the age at immigration which is relevant for classification of respondents as 1.5-generation migrants rather than 1st generation migrants. Comparing results of both commonly applied thresholds we found that it made no noticeable difference to the main associations. It was also considered to carry out separate analyses for those without children and with children as their infertility risk typically differs, but the sample size in the ethnic minority groups became too small.

Finally, we considered whether group differences in indicators of general health and lifestyle could moderate associations of migrant status with self-reported infertility. We did not include health indicators in the main analysis because they are asked in a self-completion questionnaire with much lower response rates than the main survey. Body mass index, alcohol consumption, smoking, and self-rated health were related to infertility risk but did not affect the association of migrant status and infertility.

## 4 Discussion

Based on data from a random probability sample of Australian households this study provides novel insights into the risk of self-reported infertility across migrant groups in Australia. Specifically, this study considers potential within-group variation by comparing three migrant generations and region of origin-groups with third-generation plus Australians. Infertility prevalence rates are 7.5 % for men and 11.8 % for women, which is slightly lower than the 14 % recently reported for women in the Western Pacific region (Cox et al., 2022). In global comparison this region has the second highest period prevalence (Cox et al., 2022), suggesting a high need for reproductive healthcare in Australia.

Results from this study clearly point to a migrant advantage in infertility risk. Those who immigrated themselves as adults (first generation) or children (1.5 generation) had a lower risk of self-reported infertility in comparison to third-generation plus migrants. Associations remain stable in full models adjusted for group differences related to age, social status, patterns of family formation, fertility desire, and region of residence. These findings are in line with the healthy migrant effect (HME) which suggests that international migrants often have a health advantage resulting from (self-) selection into migration. Given Australia’s emphasis on work-related migration, migrants are not a random cross-section from their home countries, but rather a selectively chosen group. The policy-driven selection based on skills and health likely contributes to the health of first-generation migrants, resulting in their lower infertility risk.

Furthermore, our analyses reveal that 1.5-generation migrants also experience a significantly lower risk of infertility than the reference group. We assume that the HME can explain the low risk of self-reported infertility for those who immigrated as children to Australia by extending the positive selection of their parents to the children born prior to their move. Parents may be more likely to move overseas when their children are also healthy in general. As in this study, a recent study on Denmark found a health advantage of first- and 1.5-generation migrants in physical and mental health outcomes (Tegunimataka, 2023). In contrast, others do not find a health advantage for 1.5 generation migrants in physical and mental health in Finland (Loi et al., 2021) and in Canada (Vang et al., 2017).

The migrant advantage occurs for all region of origin groups except for migrants from English-speaking countries who do not differ in their infertility risk from long-term residents. This may be explained by the fact that migrants from English-speaking countries face comparatively lower barriers when migrating to Australia (especially those from New Zealand) than other migrant groups. Therefore, they are likely to be less positively selected on health. By contrast, migrants from Asia and other regions experience higher migration thresholds, which tends to amplify positive health selection consistent with the healthy migrant hypothesis. Additionally, for migrants from non-ESC countries, those who actually migrate are not only selected by their health, but they also have higher socio-economic status relative to the population in their country of origin. Admittedly our groupings of regions of origin are broad due to data restrictions, likely hiding heterogeneity within these regions. This highlights the need for better data with oversampling of migrants.

Another important finding is that the migrant advantage diminishes across generations. Second generation migrants in Australia do not have a different risk of self-reported infertility from long-term residents, thus they generally do not benefit from the positive selection of their parents. Again, this result confirms results from previous studies showing that as immigrants spend more time in Australia, which is the case for second generation migrants, their health advantage dissipates (Huang et al., 2023; Anikeeva et al., 2010). Importantly with regard to infertility the health of second-generation migrations is not getting worse than that of the majority population suggesting that marginalization processes are not as strong as in other countries where such an effect can be found already for the second generation.

This study highlights the relevance of country context and in particular how access to the country is regulated in studies of migrants’ reproductive health. The majority of immigrants who enter Australia have a visa, and irregular immigration is less frequent than in Europe. By contrast, Europe grants freedom of movement within the EU territory and regulates access to EU countries primarily based on family reunion and humanitarian actions. These different selection mechanisms have implications for the composition of migrant groups in many respects including their health and its socio-demographic determinants like age and education. Consequently, for Europe, very different patterns of infertility are evidenced (Milewski et al., 2025; Brautsch et al., 2023).

One limitation of this study is that the HILDA measure of self-reported infertility conditions on medical advice. In many cases, this medical advice may result from consulting a physician about reproductive difficulties; in others, it may arise during consultations for other health conditions or medical treatments (e.g., cancer). Information about infertility status stemming from medical advice will be useful for reliably identifying cases with a medical problem. However, among minority groups, there may be worse access to health care and less frequent doctor visits. Consequently, it cannot be ruled out that these groups may be less aware of fertility problems, potentially explaining their low infertility risk. To test this better data would be needed that included different indicators of infertility.

When interpreting results from this study it is important to consider that the HILDA data is representative of migrants who arrived in Australia before 2001. The introduction of the refreshment sample in 2011 improved the representation for more recent migrants, but those who immigrated after 2001 are still more likely to be underrepresented (Summerfield et al., 2022). Nevertheless, HILDA data are among the most comprehensive and suitable data for conducting this type of analysis.

There are immigrant groups in Australia, such as family members and refugees, for whom the HME may not apply. These groups likely contribute few observations to our analytical sample, but we cannot confirm this because in HILDA the type of visa is not known for the full sample. Regarding refugees, for example, we know that at the start of the HILDA survey in 2001, they made up less than 5 % of Australia’s permanent migration program (Karlsen, 2016). Nevertheless, we would like to stress that the previous literature on reproductive health outcomes provides a heterogeneous picture, depending on the regions of origin and destination of the migrants. The type of visa used for successful entry to a country, is only one factor that may explain any group differences. Other factors include the actual main reason for migrating (which we cannot analyse) and access to and utilization of healthcare at destination. In some cases, these explanations overlap. One example is the rising share of migrants holding temporary visa who do not have access to Medicare, Australians universal health insurance scheme.

## 5 Conclusion

This study reveals a migrant advantage in infertility risk among first- and 1.5-generation migrants. This migrant advantage is driven by all origin groups except for migrants from English-speaking countries. Importantly, among second-generation migrants there are no more differences in infertility risk compared to third-generation plus Australians. The relatively high levels of self-reported infertility in this study underscore a growing need for infertility care in Australia, driven in part by the trend of delayed childbearing to older ages. Efforts to educate and inform the public about infertility risks and available reproductive health services must consider the increasing heterogeneity of Australia’s population, reflecting its status as a classic immigration country.

## Funding

This research did not receive any specific grant from funding agencies in the public, commercial, or not-for-profit sectors.

## CRediT authorship contribution statement

**Jasmin Passet-Wittig:** Writing – review & editing, Writing – original draft, Formal analysis, Conceptualization. **Ester Lazzari:** Writing – review & editing, Formal analysis, Conceptualization. **Nadja Milewski:** Writing – review & editing, Writing – original draft, Conceptualization.

## Declaration of competing interest

The authors declare that they have no known competing financial interests or personal relationships that could have appeared to influence the work reported in this paper.
